# Isolation and purification of glycosylphosphatidylinositols (GPIs) in the schizont stage of *Theileria annulata* and determination of antibody response to GPI anchors in vaccinated and infected animals

**DOI:** 10.1186/s13071-018-2651-9

**Published:** 2018-02-06

**Authors:** Toktam Abbasnia, Ahmad Asoodeh, Gholamreza Habibi, Alireza Haghparast

**Affiliations:** 1 Division of Biotechnology, Faculty of Veterinary Medicine, Ferdowsi University of Mashhad, P.O. Box: 91775-1793, Mashhad, Iran; 2 Department of Chemistry, Faculty of Science, Ferdowsi University of Mashhad, Mashhad, Iran; 3 Department of Parasite Vaccine Research and Production, Razi Vaccine and Serum Research Institute, Karaj, Iran; 4 Immunology Section, Department of Pathobiology, Faculty of Veterinary Medicine, Ferdowsi University of Mashhad, Mashhad, Iran

**Keywords:** Enzyme linked immunosorbent assay, Gas chromatography-mass spectrometry, Glycosylphosphatidylinositol, High performance liquid chromatography, Immune responses, *Theileria annulata*

## Abstract

**Background:**

Tropical theileriosis is widely distributed from North Africa to East Asia. It is a tick-borne disease caused by *Theileria annulata*, an obligate two-host intracellular protozoan parasite of cattle. *Theileria annulata* use leukocytes and red blood cells for completion of the life-cycle in mammalian hosts. The stage of *Theileria annulata* in monocytes and B lymphocytes of cattle is an important step in pathogenicity and diagnosis of the disease. Glycosylphosphatidylinositols (GPIs) are a distinct class of glycolipid structures found in eukaryotic cells and are implicated in several biological functions. GPIs are particularly abundant in protozoan parasites, where they are found as free glycolipids or attached to proteins in the plasma membrane.

**Results:**

In this study we first isolated and purified schizonts of *Theileria annulata* from infected leukocytes in *Theileria annulata* vaccine cell line (S15) by aerolysin-percoll technique. Then, the free GPIs of schizont stage and isolated GPI from cell membrane glycoproteins were purified by high performance liquid chromatography (HPLC) and confirmed by gas chromatography-mass spectrometry (GC-MS). Furthermore, enzyme linked immunosorbent assay (ELISA) on the serum samples obtained from naturally infected, as well as *Theileria annulata*-vaccinated animals, confirmed a significant (*P* < 0.01) high level of anti-GPI antibody in their serum.

**Conclusions:**

The results presented in this study show, to our knowledge for the first time, the isolation of GPI from the schizont stage of *Theileria annulata* and demonstrate the presence of anti-GPI antibody in the serum of naturally infected as well as vaccinated animals. This finding is likely to be valuable in studies aimed at the evaluation of chemically structures of GPIs in the schizont stage of *Theileria annulata* and also for pathogenicity and immunogenicity studies with the aim to develop GPI-based therapies or vaccines.

## Background

*Theileria annulata* is a protozoan parasite causing tropical and Mediterranean theileriosis in different regions around the world. Prevalence, mortality and morbidity of this disease is considerably high, which have led to serious economic deprivation due to the loss of productivity [1, 2].

*Theileria annulata* is an obligate unicellular parasite that has two hosts (vertebrates and invertebrates) which is transmitted by *Hyalomma* ticks; the infection in ticks is typically established by feeding in an infected vertebrate host for 48–72 h [3–5]. When an infected tick is feeding on cattle, sporozoites of *T. annulata* are inoculated into the blood from salivary glands of the tick. After sporozoites invade leukocytes (B lymphocytes and monocytes) they proliferate and transform to macroschizont, microschizont and finally merozoites in the infective leukocytes. Merozoites released from leukocytes, invade erythrocytes and develop into piroplasm, which is the final stage in the vertebrate host [6–8]. It has been shown that the most important signs of theileriosis are caused by immortality and lymphoprolifration of leukocytes due to the schizont stage of *T. annulata* [6].

Several proteins and glycoproteins that are involved in induction of immune responses of the host have been found on the outer membrane surface of schizont [9, 10]. Recent studies suggested that glycosylphosphatidylinositols (GPIs) of protozoan parasites may also be involved in the generation of host immune responses [11].

**Fig. 1:**
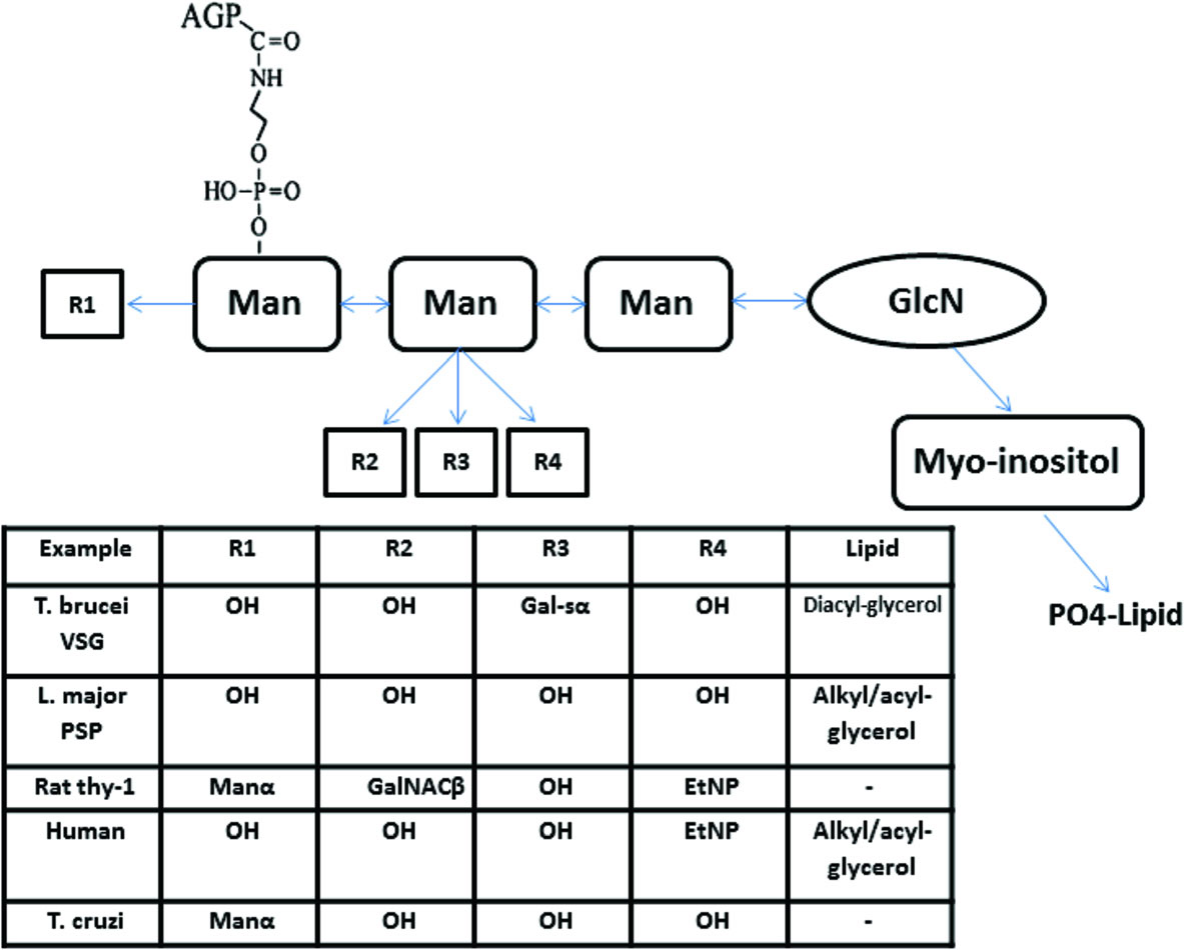
Common structure of GPI molecules in many eukaryotic cells

GPIs are glycolipid structures that are ubiquitously expressed in the membrane of eukaryotic cells. GPIs have various functions and structures, and some of these molecules anchor proteins on the cell membrane. The GPIs anchor is a post-translational modification and the modified protein is anchored on the outer surface of the cell membrane. GPIs have a complex structure that includes a phosphoethanolamine linker, glycan core and phospholipid tail (Fig. 1). The phosphoinositol, glucosamine, mannose residues and other sugars can be seen within the glycan core. This complex structure of GPIs suggests that this molecule may probably have diverse functional capacity beyond membrane insertion [12, 13].

In different organisms, GPIs differ in their acyl/alkyl substituents in phospholipid tail, having additional sugar moieties on the first, second or third mannose, extra ethanolamine phosphate groups on the core glycan structure, and an acyl substituent on C-2 of inositol. GPIs have many different biological functions that are partly due to diversity in their structures. Many parasitic protozoa synthesize GPIs in excess of the amount required to anchor outer membrane proteins to the cell membrane. These GPIs are likely to play important roles in properties of cell membranes and the modulation of immune responses in the hosts [13, 14]. Structural studies have shown that the complexity and diversity of GPIs structures is greater in the protozoa cells compared to mammalian cells [14]. Apicomplexan protozoa are a phylum of parasites, and GPI structure is vital for the life-cycle of these organisms [15–17]. GPIs of several apicomplexan protozoa including *Plasmodium falciparum*, *Toxoplasma gondii* and *Trypanosoma cruzi* have already been characterized [18].

The main immunological function attributed to GPIs of *Plasmodium falciparum*, *Toxoplasma gondii* and *Trypanosoma cruzi* are the comparable induction of pro-inflammatory cytokines, including tumor necrosis factor alpha (TNF-α), interleukin 12 (IL-12), gamma interferon (IFN-γ) and IL-6 secretion [19–21].

Similar to other pathogens, macrophage activation and pro-inflammatory cytokines production in the protozoan infected host cells involve the activation of cells by the pathogen associated molecular patterns (PAMPs) via host cell innate immunity sensors, the most important of them, toll like receptor 2 (TLR2) and TLR4 [22, 23]. A monoclonal antibody against *P. falciparum* GPIs has been reported to neutralize the TNF-α inducing activity of GPIs, suggesting that naturally elicited anti-GPI antibodies can provide protection against malaria pathogenesis [18, 24].

In a recent study, three synthetic GPI analogues of *P. falciparum* were conjugated to keyhole limpet haemocyanin (KLH) as a carrier protein and injected them into mice. Antibodies generated against synthetic GPI-protein were able to recognize native GPI of *P. falciparum*, suggesting that GPI as a candidate molecule for rapid diagnosis in malaria [25].

Focusing on the detailed structures of schizont stage of *T. annulata* is the prime focus of current research and, as already mentioned, the schizont stage of *T. annulata* is the most prominent stage in parasite invasion and pathogenicity [26]. As yet, there is no information available regarding the isolation, characterization and biological function of GPIs of *T. annulata*. Therefore, in the present study, we aimed to isolate the GPIs of schizont stage of *T. annulata* and confirm the presence of GPI structure by gas chromatography-mass spectrometry (GC-MS). Moreover, in order to evaluate the immunological properties of the purified GPIs, the presence of specific antibodies against GPIs molecules in the serum of vaccinated and infected animals was detected by enzyme linked immunosorbent assay (ELISA).

## Methods

### Parasite strain, culture conditions and purification

The vaccine cell line, strain S15 Iran, of *T. annulata* used in this study was obtained from the Razi Vaccine and Serum Research Institute (Karaj, Iran). The parasites were cultured in RPMI-1640 medium (Sigma-Aldrich, Darmstadt, Germany) supplemented with 10% FBS (Sigma-Aldrich, Darmstadt, Germany), 292 μg/ml L-glutamine (Sigma-Aldrich, Darmstadt, Germany), 4.5 mg/ml glucose, 100 μg/ml penicillin (Sigma-Aldrich, Darmstadt, Germany), and 100 μg/ml streptomycin (Sigma-Aldrich, Darmstadt, Darmstadt, Germany). Schizonts were purified as described by Baumgartner et al. [27]. Briefly, S15 cells were incubated for 4 h with 3 μM nocodazole to depolymerize the microtubules of host cell. Cells were then treated with aerolysin on ice for 30 min. After removing excess aerolysin with ice cooled phosphate buffered saline (PBS), cells were incubated at 37 °C for 30 min to stimulate toxin-mediated permeabilization of the host cell plasma membrane. Permeabilization was monitored using Trypan blue exclusion assay. Host cell debris and nuclei were separated from schizonts of *T. annulata* by Percoll gradient centrifugation. A stock solution of Percoll was prepared by mixing 8.5 parts of Percoll with 0.5 part of 20× HEPES (200 mM HEPES, 3 M NaCl, 400 mM KCl, pH 7.4) and one part of 50 mM EDTA (pH 7.4). In this study, ultracentrifugation was not used so a solution in 2 ml microtubes was made. The cell lysate (138 μl) was added to 520 μl of this Percoll stock solution and the volume was adjusted to 680 μl by the addition of 1× HEPES containing 5 mM EDTA, giving rise to 64.6% (vol/vol) final Percoll concentration. The Percoll-cell lysate mixture was transferred into 2 ml microtubes and carefully overlaid with a 45% Percoll solution in 1× HEPES and 5 mM EDTA. The mixture was thereafter centrifuged at 12,000× *rpm* for 1 h at 10 °C. During centrifugation, a gradient is established that separates parasites by density from cellular debris, nuclei and purified schizonts, and after centrifugation, we observed two bands, one of which was purified schizonts. These bands were collected with a Pasteur pipette. Next, the purified schizonts parasites layer was collected in a 2 ml microtube. Percoll crystals, which could be seen in Giemsa staining in this stage, were removed by washing once in a large volume of PBS and pelleted by centrifugation at 5000× *rpm* for 10 min at 4 °C. Purified schizonts were visualized under a light microscope by Giemsa staining and with no nuclei of DAPI (4,6-diamidino-2-phenylindole).

### Purification of GPI anchors of schizonts

GPI was isolated from schizonts of *T. annulata* as described hereafter. After purification of schizonts from 5 × 10^10^ host cells, they were lyophilized and re-suspended in ice-cold chloroform/methanol/water 8:4:3. Afterwards, they were centrifuged and supernatants were collected. For each 6 ml of the extracted supernatants, 5.6 ml of deionized water were added. The upper methanol/water-rich phase contained most of the glycolipids and GPIs. The upper phase was dried under the nitrogen environment. After drying, 2 ml of water and 2 ml n-butanol were added, vortexed and separated in two phase. The upper butan-1-ol rich phase was taken and an equal volume of pre-equilibrated lower phase was added, vortexed and centrifuged to separate the phases. This step was repeated twice. The upper phase (butan-1-ol rich phase) was taken and dried and then stored under nitrogen steam. This fraction is highly-enriched in GPIs and other glycolipids.

### Purification of schizont glycoproteins

Glycoproteins from schizont stage of *T. annulata* were purified using a slight modification of previous procedures [27, 28]. Briefly, parasite pellets containing a total of 2 × 10^10^ schizonts were freeze-dried and sequentially de-lipidated with 50 ml of each of the following solvent mixtures: (i) chloroform/methanol (2:1, *v*/v); (ii) chloroform/methanol (1:1, v/v); (iii) chloroform/methanol (1:2, v/v); and (iv) chloroform/methanol/water (10:10:3 by v/v). Between each extraction step, insoluble cell debris was separated from the organic phase by centrifugation (1500× *g*, 15 min, 10 °C). The final de-lipidated parasite debris and supernatant were dried under N_2_ separately. The dried supernatant was dissolved in butan-1ol /water (2:1 v/v) for 4 h in room temperature and the dried pellet was dissolved in 9% butan-1-ol for 4 h at room temperature.

Butanolic phase of supernatant and upper phase of cell debris were pooled. The resulting extracts were combined, dried under vacuum, dissolved in 5% propan-1-ol, and 0.1 M ammonium acetate (buffer A), and then applied onto an Octyl-Sepharose (GE Life Science 83,264, Freiburg, Germany) column (1.0 × 10 cm) at a flow rate of 4 ml/h, at room temperature. After washing with 10 volumes of buffer A and 10 volumes of 5% propan-1-ol, the column was eluted using a propan-1-ol gradient (5–60%) at a flow rate of 12 ml/h. Twenty fractions were collected. Up to 5 μl aliquots of each fraction were used for glycoproteins analysis by sodium dodecyl sulfate polyacrylamide gel electrophoresis (SDS-PAGE).

### Isolation of GPI anchor from glycoproteins

Fractions (50 μl) that were detected by SDS-PAGE staining were dried in a SpeedVac, re-dissolved in 90 μl of 10 mM Tris–HCl pH 7.8, and 1 mM CaCl_2_, and incubated with 10 μl of proteinase K (5 mg/ml; Sigma-Aldrich, Darmstadt, Germany) for 16 h at 37 °C. The incubation was terminated by heating at 100 °C for 5 min. The sample was then extracted three times with 200 μl of water-saturated butan-1-ol (91% butan-1-ol). The released GPI moiety was recovered in the butanolic phases, which were combined and washed further (three times) with water to remove any residual glycopeptides and/or salts. GPI anchor was then freeze-dried [29].

### HPLC purification of GPI anchors and glycan core

Components of dried butanolic phases were separated by chromatography on reverse-phase high performance liquid chromatography (RP-HPLC) with a semi-preparative C8 column (10 mm × 250 mm), manufactured by Macherey-Nagel GmbH & Co., Duren, Germany) using a linear gradient of 20–60% aqueous 1-propanol containing 0.1% trifluoroacetic acid (TFA) over a period of 80 min and held for 30 min at a flow rate of 0.5 ml/min [30]. Samples corresponding to the four peaks were collected.

Aliquots of HPLC collected peaks (100 μl) were delipidated, dephosphorilated, de-aminated and subsequently reduced. Briefly, samples were de-lipidated by base hydrolysis in concentrated ammonia (35% *w*/w in water, Fisher chemical, A/3295/PB05):methanol (1:1), and incubated at 50 °C for 6 h. The base was removed by N_2_ stream, and dried twice to remove residuals. Dephosphorylation was performed by 48% HF for 60–72 h at 0 °C. The reaction was stopped by freeze drying [31, 32].

For de-amination and reduction, each sample re-dissolved in 30 μl 300 mM sodium acetate buffer (pH 4.0). Freshly prepared 1 M sodium nitrite (30 μl) was added to each sample and incubated for 3 h at room temperature. Then 15 μl freshly prepared 0.8 M boric acid was added. Afterwards, 16 μl 1 M NaOH and 23 μl NaBD4 1 M (Sigma-Aldrich, Milwaukee, WI, USA) were added and incubated at room temperature for 3 h [31, 32].

### **High performance thin layer chromatography (HPTLC)** analysis of HPLC peaks and SDS-PAGE glycoproteins bands

HPLC peaks and 35% propan-1-ol band of glycoproteins in SDS-PAGE analysis were applied onto HPTLC. Silica gel 60 F254 was purchased from Merck (Darmstadt, Germany). The plate was developed with C/M/W (65/35/8), dried and stained with orcinol, water, ethanol and H_2_SO_4_.

### Myo-inositol and monosaccharide derivatization

Twenty μl of 1 M Scyllo-inositol (Sigma-Aldrich, Darmstadt, Germany) was added to each samples as internal standard and hydrolyzed in dry methanol by 6 M HCl for myo-inositol and 0.5 M HCl for mannose and GlcNA. Finally, 15 μl of fresh trimethylsilylderivatization (TMS) reagents containing trimethylchlorosilane/hexamethyldisilazane (Sigma-Aldrich, Darmstadt, Germany)/dry pyridine 50 μl:150 μl: 1 ml were added [31, 32].

### Gas chromathography mass spectrometery (GC-MS)

Samples were analyzed using a Varian GC-MS system (Palo Alto, California, USA) equipped with DB-5 capillary column (30 m length × 0.25 mm diameter × 0.25 μm film thickness). Helium was used as the carrier gas at a constant flow rate of 1.3 ml/ min. The injector and MS source temperatures were constant at 140 °C and 260 °C, respectively. The oven temperature was programmed from an initial temperature 140 °C for 2 min, then temperature gradient elevated to 250 °C at 6 °C/min, hold for 15 min at 250 °C, followed by holding at 300 °C for 15 min [31, 32].

### Detection of anti-GPI antibody response in vaccinated and infected animals

To examine the presence of anti-GPI antibodies in serum of vaccinated cattle, sera were taken from three cattle, thirty days after vaccination with the attenuated cell line vaccine (S15). Serum of seven naturally infected cattle with confirmed theileriosis was also obtained. Confirmation of the infection was based on positive peripheral blood smear test (detection of piroplasm stage of the parasite in peripheral blood smear stained with Giemsa) and PCR technique (the specific primers were designed based on *T. annulata* 18S ribosomal RNA gene sequence). For negative control, four newborn calf serums and four human serums were used. In ELISA, 44 nmol/well HPLC purified GPIs were coated onto 96-well microtiter plates (Greiner Bio-One GmbH, Frickenhausen, Germany) and incubated at 37 °C overnight. After washing, the wells were blocked with 2% bovine serum albumin (BSA) (Sigma-Aldrich, Darmstadt, Germany) in PBS, and after 1 h incubation at 37 °C, diluted sera (1/10,000 and 1/5000) of vaccinated cattle were added in triplicates wells. For a negative control, newborn calf serum and human serum were added in triplicate wells. The bound antibodies were measured by HRP-conjugated goat anti-cow IgG (Abcam ab102154, Cambridge, USA). For measuring bound antibodies in human sera, HRP-conjugated goat anti-human IgG (Abcam ab102420, USA) was used. We were unable to obtain any positive GPI control serum samples in this experiment. In some wells, PBS instead of GPI (Ag) was coated and serums of vaccinated, infected and newborn calf were used in triplicates as control. The reaction was visualized using 100 μl tetramethylbenzidine (TMB) as substrate*.* For stopping the reaction 100 μl of sulfuric acid (H_2_SO_4_) was added to each well and optical density (OD) was read at 450 nm in an ELISA plate reader (ELX800 absorbance reader, BioTeK, Winooski, USA).

### Statistical analysis

All data were analyzed using Prism 6.01 (Graphpad, La Jolla, CA, USA) software. Statistical significance of differences between groups was determined using one-way ANOVA. *P*-values < 0.01 were considered significant.

## Results

### Isolation and purification of GPI from purified schizont and HPLC analysis of n-butanol extracts

**Fig. 2:**
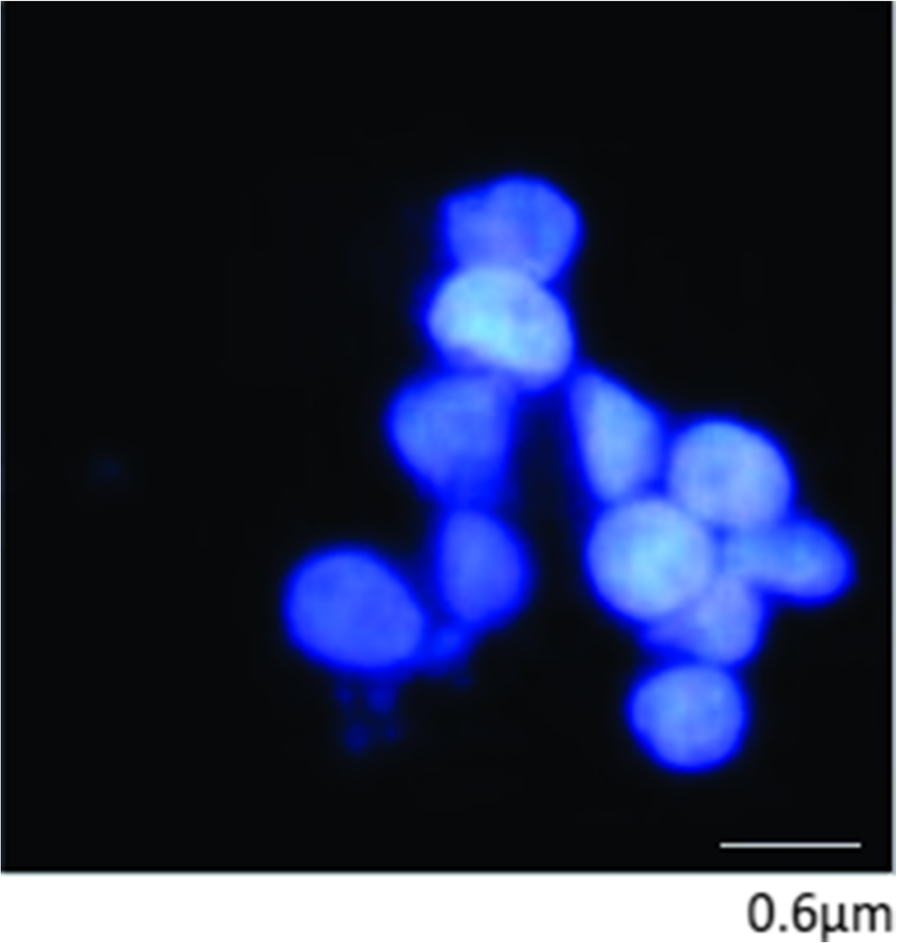
DAPI staining of purified schizont of *T. annulata* free of host nuclei under UV light (magnification 1000×). *Scale-bar*: 0.6 μm

**Fig. 3:**
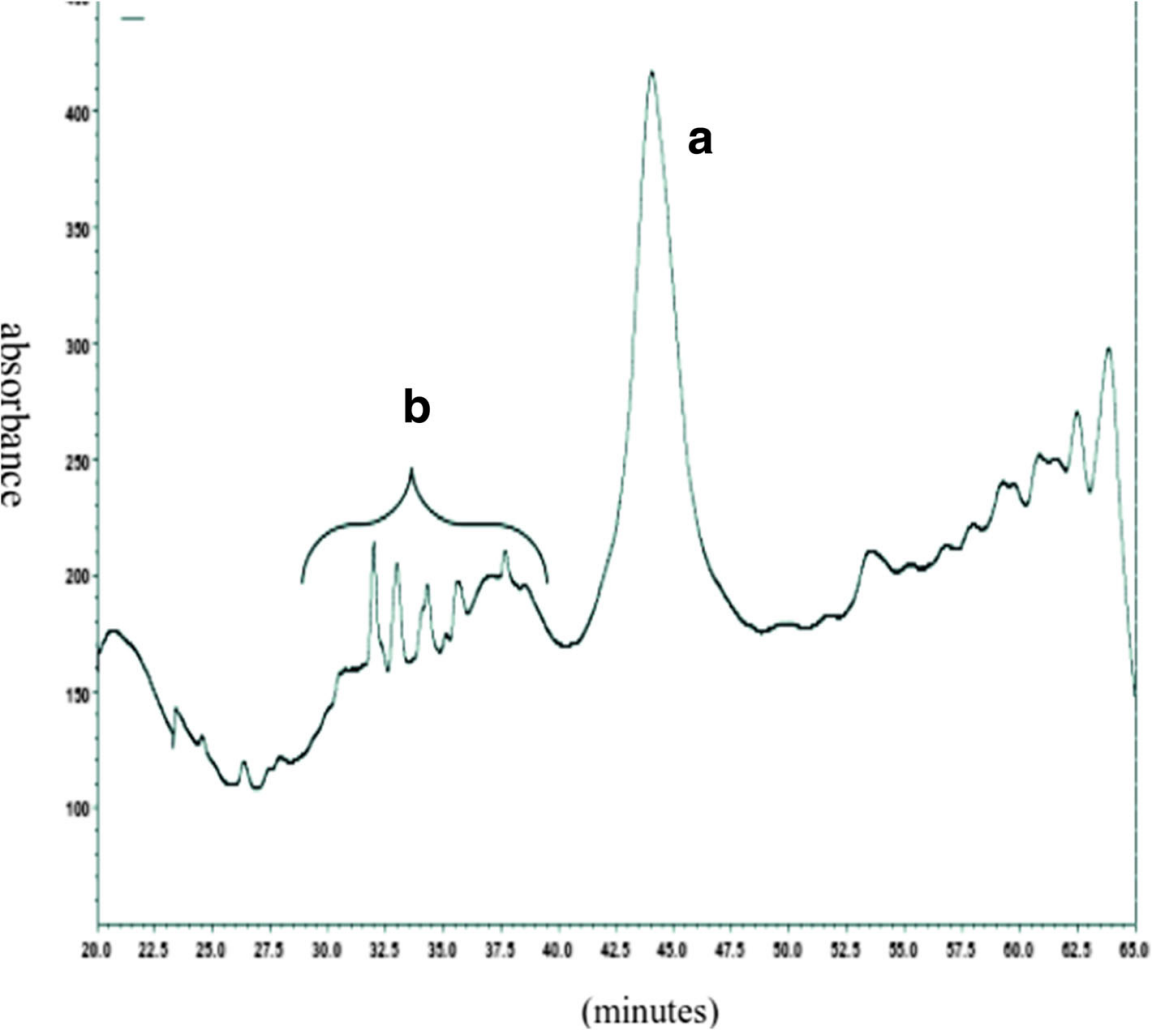
HPLC diagram of n-butanol extracted samples. The (**a**) peak in the 44 min of isolation belongs to GPI according to GC-MS and HPTLC analysis. The (**b**) peaks of the other glycolipipids of schizont stage of *T. annulata*

*Theileria annulata* and its host cells is eukaryotic organism and both of them have GPI in their cell membranes. 5 × 10^10^ of S15 Iran vaccine strain was cultured and grown to density of approx.8–10 × 10^5^/ml. The quality of purified schizont in aerolysin-gradient centrifuge percoll technique was 10%. Parasite pellet contained in total 5–8 × 10^10^ purified schizonts. Purified schizonts were visualized under the light microscope by Giemsa staining and with no nuclei of host cells in stained purified schizonts (Fig. 2). DAPI (4,6-diamidino-2-phenylindole) is a blue fluorescent nucleic acid stain that binds strongly to A-T rich regions in DNA. DAPI staining visualized large nucleus of host cells and nuclei of *T. annulata* schizonts of S15 Iran vaccine strain. In DAPI staining of purified schizonts, large nucleus of host cell could not be seen next to the nuclei of schizont (Fig. 2). GPI molecules were differentially extracted to remove non-glycosylated lipids, and therefore the extract contained the free GPI and glycoproteins. Finally, the GPIs of *T. annulata* schizonts cell membrane were purified by successive fractionation using HPLC. The GPIs that are linked to schizont proteins were isolated with octylcepharose chromathography after exhaustive digestion of the delipidated glycoprotein of parasites with proteinase K. In this chromatography only GPI-proteins bond to columns and other glycoproteins exit from columns by washing buffers. Six major peaks between 30 and 50 min were collected for more analysis by GC-MS (Fig. 3).

### HPTLC analysis of carbohydrate

**Fig. 4:**
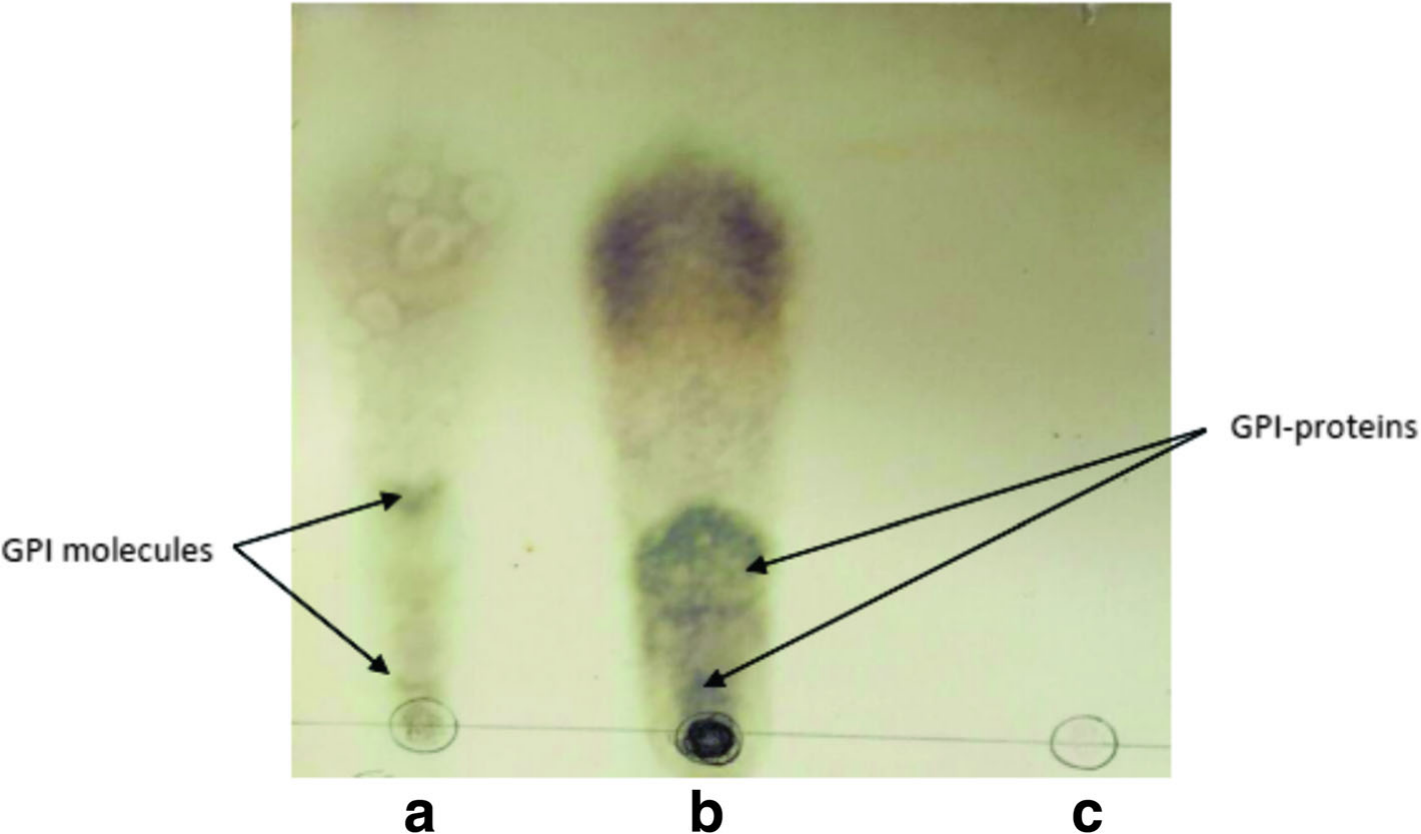
HPTLC picture of GC-Mass analysis of GPI molecules of *T. annulata* schizont stained with specific stain of carbohydrates (orcinol-sulfuric acid) are shown. **a**: Peak of 44 min in HPLC. **b**: 35%propan-1-ol band of glycoprotein of octylcepharose test in SDS-PAGE. **c**: Albumin as negative control

Orcinol-H_2_SO_4_ staining in HPTLC analysis is specific for carbohydrate (sugar-like mannose, galactose, glucose, ribose etc.) As shown in Fig. 4, a sample of glycoprotein in 35% propanol of octylcepharosestaind with orcinol-sulphoric acid (Fig. 4a). In Fig. 4b, a 44 min peak of HPLC stained with specific stain for carbohydrate (orcino- sulphoric acid) is shown. The results of HPTLC showed carbohydrates isolated in earlier stages whose main components of GPI in core glycan.

### GC-MS analysis of GPI glycan core

**Fig. 5:**
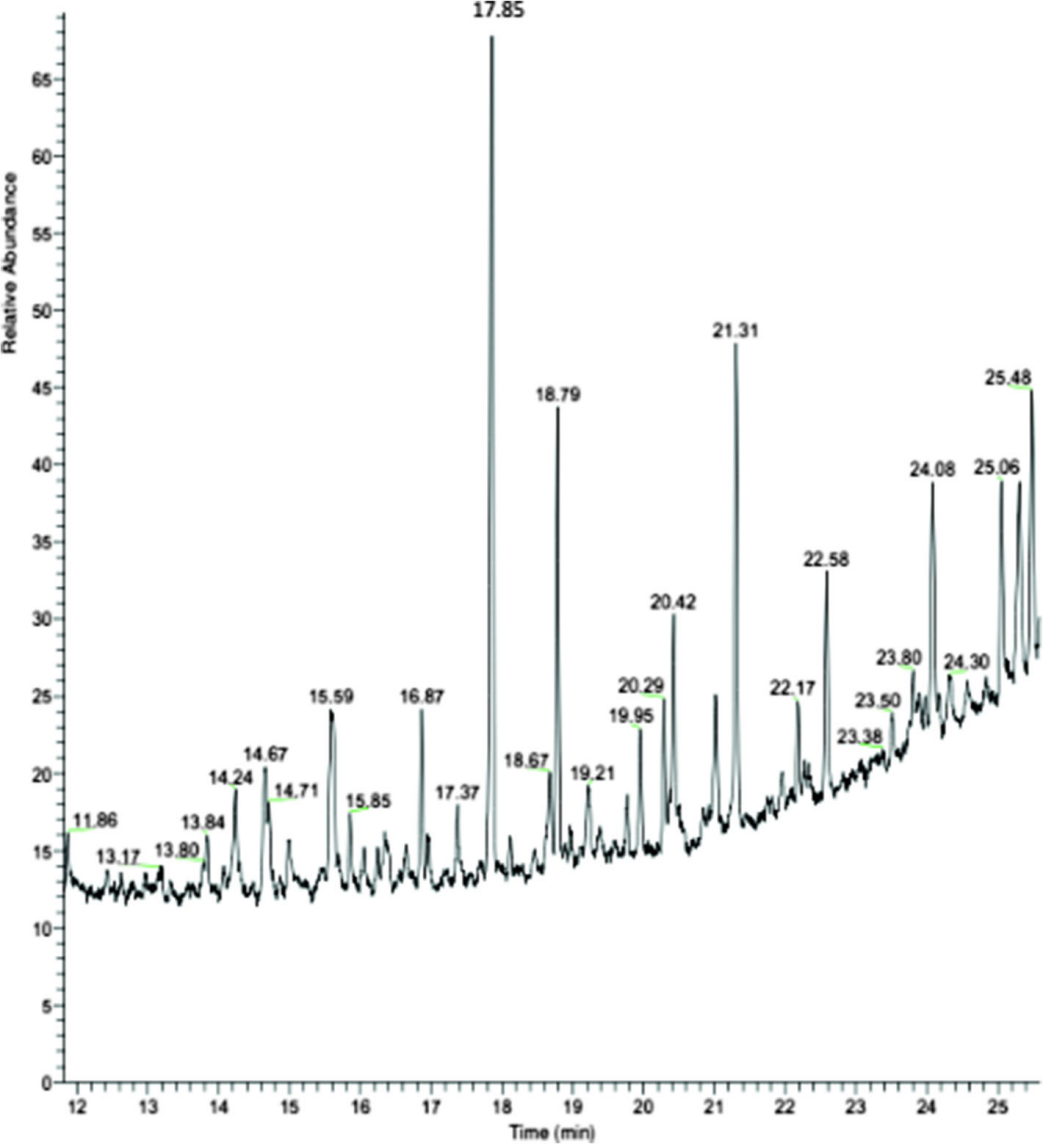
GC-Mass analysis of GPI molecules of *T. annulata* schizont. Total ion chromatogram (linear scanning m/z 40–650) of the AHM, mannose, glucose, galactose, ethanolamine and inositol TMS derivatives from GPI molecules of *T. annulata* schizont are shown. AHM, 24.08 min; mannose, 18.11 min; Gal, 17.85 min; Glc, 15.6 min, Scyllo inositol, 17.37 min; myo-inositol, 19.21 min; ethanolamine, 15.58 min

For GC-MS analysis, this was followed by tri-methyl-silyl(TMS)derivatization of monosaccharides and myo-inositol. In TMS derivatization, functional groups of the components that do not vaporize at temperature below 350–400 °C, are chemically modified. The structure EtN-*P*-Man4-GlcN-IP-G is prepared by hydrolysis with 35% ammonia in aqueous methanol. The acylated-inositol carbohydrate moiety with and without the phosphate group on inositol ring (Man4-GlcN-acyl-IP and Man4-GlcN-acyl-Ino) and carbohydrate moiety (OCH-CH2-*P*-Man4-anhydromannitol) that lacked the PI residue of GPIs were prepared by HF cleavage and nitrous acid deamination, respectively. Tri-methyl-silylderivatization of monosaccharides and myo-inositol functional groups before GC-MS analysis increases sample volatility, improves selectivity and enhances detectability. Typical elution time of analytes of glycan core in GC-MS spectrometry was as follows (Fig. 5): AHM, 24.08 min; mannose, 18.11 min; Gal, 17.85 min; Glc, 15.6 min, Scyllo-inositol, 17.37 min; myo-inositol, 19.21 min; ethanolamine, 15.58 min.

### Antibody response to GPI molecules of *T. annulata* schizont

**Fig. 6:**
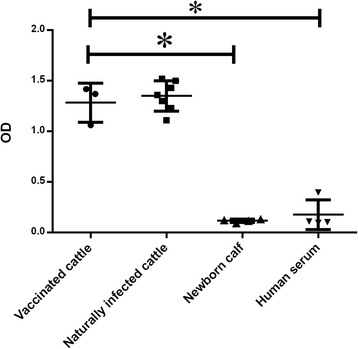
Antibody responses to GPI molecules of *T. annulata* performed by ELISA. Total IgG were measured in the serum to determine the specific antibody responses against GPIs molecules of the schizont stage of *T. annulata*. Statistical analysis was performed by one-way ANOVA (*P < 0.01). All serum samples (vaccinated, naturally infected and negative control serums) were measured in triplicates

In order to determine whether GPIs of *T. annulata* induce naturally elicited anti-GPI antibodies, parasite glycolipids were isolated and purified by sequential (chloroform/methanol) extraction and water/n-butanol phase partition and analyzed by HPLC, HPTLC and GC-MS. Afterwards, sera from naturally infected cattle (*n* = 7) as well as vaccinated cattle (*n* = 3) were analyzed 30 days after vaccination. In ELISA performed with a coating of 44 nmol/well of HPLC purified GPIs, the sera of vaccinated cattle and *T. annulata* naturally infected cattle sera showed significantly (ANOVA: *F*_(3,14)_ = 106.8, *P* < 0.0001) high levels of GPI specific antibodies (IgG), whereas all newborn calf and human sera completely lacked anti-GPI antibodies (Fig. 6).

## Discussion

*Theileria annulata* is an apicomplexan parasite that infects leukocytes of cattle. Further study on the unknown structures of parasites, and also developing new strategies to uncover the molecular structures involved in parasite biology, is of great importance. This can help to gain insight in pathogenicity and immunogenicity studies of the parasite with the aim for development of novel therapies or vaccines.

This study was undertaken to reveal the molecular structures of GPI of the schizont stage of *T. annulata*, an important protozoan parasite of cattle. In our study, the schizont stage of the parasite was purified from the host cells prior to GPI isolation from the schizont stage in order to avoid any contamination of host cell structures during GPI isolation and purification. The most important finding of this study was to establish, for the first time, *T. annulata* GPIs by direct biochemical and GC-MS analysis.

Based on these structures, a consensus structure can be postulated to show the conserved and variable features of GPIs. Recent studies have focused on specific strategies of GPI anchors that can help to better understanding of the GPI structure. Naturally, these strategies have extensively borrowed from the techniques that are used to study GPIs of other protozoan parasites [15, 19, 32, 33]. In this study, we used analytical methods that are appropriate for obtaining highly purified GPIs from relatively small amounts of starting material (1–150 nmol). The techniques relied on the use of GC-MS and liquid chromatography.

In protozoan parasites, free GPIs play important role in parasite-host immune system interaction. To date, details of GPI structure of several protozoan parasites have already been characterized. A number of studies have suggested that GPIs of *Plasmodium falciparum* are a major factor that contributes to pathogenesis and protective immunity against parasite through its ability to induce pro-inflammatory cytokines and antibody responses [34–36]. A useful parameter in cerebral malaria is the low ratio of anti-inflammatory cytokine IL-10 to pro-inflammatory cytokine TNF-α and low level of anti–GPI antibody in plasma [22, 34, 36]. *Plasmodium falciparum* GPI can insert into the non-parasitized RBC membrane, after recognition of RBC-GPI with circulatory anti-GPI antibodies, suggesting that RBC-GPI-antibody elimination may contribute to anemia in malaria [37].

GPIs are critical for *Trypanosoma* survival and this can help to evade host immune response. A study to evaluate the role of GPIs in *T. brucei* showed that GPI-anchored proteins are vital in life-cycle of *T. brucei* [38, 39] and promote the induction of pro-inflammatory cytokines responses [20, 40].

In *Toxoplasma gondii*, virulent and avirulant strains differ significantly in GPI cell contents and in their GPI biosynthesis, but induce similar levels of TNF-α and IL-12P40 secretion and initiated TLR4/MyD88 dependent NF-κBp65 signaling in monocytes and macrophages [41].

Götze et al. [42] conjugated two major GPI glycans of *T. gondii* to carrier protein and examined the protective properties of the glycol-conjugation in the mouse model against the virulent strain of *T. gondii.* Antibodies raised and bind to the respective GPIs on carbohydrate microarray, but this immune response was unable to protect mice when challenged with a lethal dose of the virulent *T. gondii* [42]*.* In other studies, highly purified GPI from tachyzoite of *T. gondii* could induce TNF-α production in macrophages of the host and it was suggested that TLR2 and to a lesser extend TLR4 may participate in host immune responses to GPIs of *T. gondii* [21, 23, 43]. The detailed molecular structures, as well as pro-inflammatory properties, of GPIs of *T. annulata* remain to be tested in further experiments.

Many GPI-anchor proteins have been identified in *T. annulata*, which was suggested to be involved in parasite-host cell interaction and immune responses of host cells [10, 11] However, in contrast to *P. falciparum*, no specific detail about the structure and biosynthesis of GPI-anchor is known in *T. annulata*. Glycan core production is the first stage for GPI analysis with GC-MS. Glycan core is linked to a phospholipid tail through myo-inositol.

The presence of a single non N-acetylated glucosamine residue in all GPI structures was confirmed in our study. A useful property of non-N acetylated GlcN is its ability to react with nitrous acid generated in situ under mild conditions (pH 4.0, room temperature) and to be almost quantitatively converted to 2,5-anhydromannose (AHM). This structure, which was present in our GC-MS analysis, is a unique component of GPI in GC-MS analysis which can confirm the GPI existence [33]. The presence of carbohydrates in the extracted HPLC product was also confirmed by HPTLC analysis (Fig. 4).The GC-Mass analysis of purified GPI of schizont stage of *T. annulata* demonstrated the presence of myo-inositol, mannose, ethanolamine, glucosamine and AHM (a unique component of GPIs) (Fig. 5). Another important finding in this study was to show that vaccinated and infected cattle to *T. annulata* developed specific anti-GPI antibody response, whereas calves that were not exposed to *T. annulata* did not show the presence of this antibody in their serum (Fig. 6).

Previous studies have shown that *P. falciparum* GPIs can induce strong antibody response in people exposed to malaria, and determined that anti-GPI antibody responses correlated with protection against malaria [25, 35]. The data presented in study of Mbengue et al. [36] in Senegal demonstrated that total anti-GPI antibody were significantly higher in surviving severe malaria patients than in fatal mild severe cases and some degree of protection for people in endemic areas is correlated with anti-GPI antibody in sera.

Strong antibody response in ELISA test in our experiment points to the presence of specific antibodies against GPI anchors in the sera of vaccinated cattle, indicating the immunogenicity properties of GPI during the schizont stage of *T. annulata* in cattle monocytes and lymphocytes.

## Conclusions

Taken together, to the best of our knowledge, this is the first report of isolation and purification of GPIs from *T. annulata* and determination of the presence of anti-GPIs antibodies in infected and vaccinated animals. This information extends our knowledge about parasite biology and specific molecules that stimulate the host immune system during protozoan infection. The identification of receptor(s) and signaling pathways triggered by these GPI-related structures may provide new insights for the development of novel vaccines, adjuvants, therapeutics and diagnostic tools that prevent detrimental immune responses observed during infection with this protozoan parasite.

## Abbreviations

ELISA: Enzyme linked immunosorbent assay
GC-MS: Gas chromatography-mass spectrometry
GPI: Glycosylphosphatidylinositol
HPLC: High performance liquid chromatography
HPTLC: High performance thin layer chromatography
KLH: Keyhole limpet haemocyanin
PAMPs: Pathogen associated molecular patterns
TLR: Toll like receptor
